# Serial type-specific human papillomavirus (HPV) load measurement allows differentiation between regressing cervical lesions and serial virion productive transient infections

**DOI:** 10.1002/cam4.473

**Published:** 2015-05-20

**Authors:** Christophe E Depuydt, Jef Jonckheere, Mario Berth, Geert M Salembier, Annie J Vereecken, Johannes J Bogers

**Affiliations:** 1 Department of Molecular Diagnostics, AML, Sonic HealthcareAntwerp, Belgium; 2 Department of Immunology, AML, Sonic HealthcareAntwerp, Belgium; 3 Laboratory for Cell Biology and Histology, University of AntwerpAntwerp, Belgium

**Keywords:** Cervical intraepithelial neoplasia, latency, liquid-based cytology, primary cervical cancer screening, real-time quantitative PCR

## Abstract

Persistent high-risk human papillomavirus (HPV) infection is strongly associated with the development of high-grade cervical intraepithelial neoplasia (CIN) or cancer. Not all persistent infections lead to cancer. Viral load measured at a single time-point is a poor predictor of the natural history of HPV infections. However the profile of viral load evolution over time could distinguish nonprogressive from progressive (carcinogenic) infections. A retrospective natural history study was set up using a Belgian laboratory database including more than 800,000 liquid cytology specimens. All samples were submitted to qPCR identifying E6/E7 genes of 18 HPV types. Viral load changes over time were assessed by the linear regression slope. Database search identified 261 untreated women with persistent type-specific HPV DNA detected (270 infections) in at least three of the last smears for a average period of 3.2 years. Using the coefficient of determination (*R*²) infections could be subdivided in a latency group (*n* = 143; *R*² < 0.85) and a regressing group (*n* = 127; *R*² ≥ 0.85). In (≥3) serial viral load measurements, serial transient infections with latency is characterized by a nonlinear limited difference in decrease or increase of type-specific viral load (*R*² < 0.85 and slopes between 2 measurements 0.0010 and −0.0010 HPV copies/cell per day) over a longer period of time (1553 days), whereas regression of a clonal cell population is characterized by a linear (*R*² ≥ 0.85) decrease (−0.0033 HPV copies/cell per day) over a shorter period of time (708 days; *P* < 0.001). Using serial HPV type-specific viral load measurements we could for the first time identify regressing CIN2 and CIN3 lesions. Evolution of the viral load is an objective measurable indicator of the natural history of HPV infections and could be used for future triage in HPV-based cervical screening programs.

## Introduction

Although effective cytology screening methods were introduced in the previous century, today more than halve a million women are still diagnosed with cervical cancer each year and about 275,000 women die from it 1. At the end of the 20th century, it was discovered that virtually all cervical cancer cases are caused by a persistent human papillomavirus (HPV) onco-protein driven expression in infected basal cells. These infected basal cells evolve to precursor lesions detectable by screening 2, which can be treated avoiding progression to an invasive cancer 3–6. We recently showed that the development of cervical precancer (cervical intraepithelial neoplasia of grade 3 [CIN3]) is preceded by a steady increase in the viral load of a given HPV type (transforming process), whereas a rapid exponentially increasing load (virion producing transient infection) is generally cleared within 6–18 months and is usually associated with low-grade cytological abnormalities 7. The standardized quantification of the linear slope of the increase in viral load could predict incipient CIN3 lesions long before cervical cancer would occur.

Randomized trials have demonstrated that a HPV-based screening leads to a significant decrease in the incidence of CIN3 and cancer 8 and these findings provide the evidence to recommend HPV testing rather than cervical cytology as preferred screening test.

**Figure 1:**
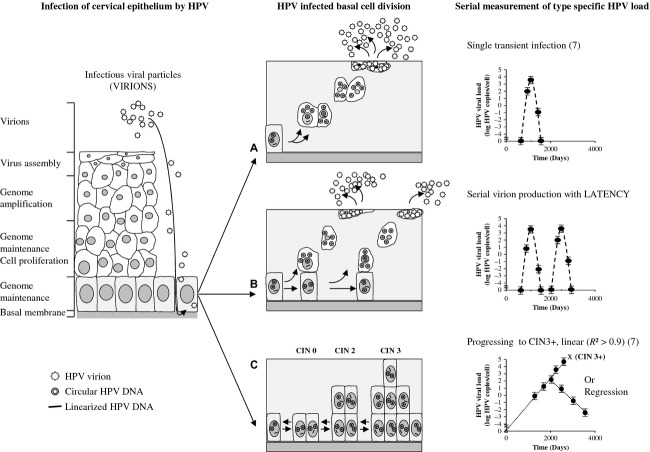
Categorization of HPV infection according to infected basal cell division. In this model 3 different possibilities were considered for basal cell division, with division leading to (A) 2 parabasal cells, (B) 1 parabasal and 1 basal cell (asymmetric replacement), and (C) doubling of HPV infected basal cells. CIN, cervical intraepithelial neoplasia; black circles, viral load measurements; Dashed lines, virion producing episode. Triangle represents the calculated starting point of the linear increase leading to CIN3+ with viral load of -5 log HPV copies/cell. The dotted line represents the least squares line. X, detection of CIN3+ 7.

Ultimately, the implementation of primary HPV screening will result in earlier detection of cervical cancer and later on of its precursors, increasing the clinical sensitivity. The implementation of HPV screening and increased coverage of HPV vaccination will lower the background risk of CIN3 and cancer, leading to longer screening intervals and a requirement for more specific tests 9. Therefore, type-specific serial measurements with a more sensitive and more specific HPV assay would allow earlier detection and treatment 7,10. Hybrid capture II (HC2) and most other HPV assays currently validated for use in primary cervical cancer screening may not be able to fulfill this optimal purpose 10. One of the unknown parameters of HPV natural history hampering primary HPV screening is the understanding of viral latency 11,12 as well as the regression of HPV induced lesions 13,14. Recently, we described a new categorization of HPV infections based on serial measurements of type-specific viral load, and proposed an underlying mechanism based on differential cervical basal cell division after infection (Fig.1). Under this model three different possibilities were considered for basal cell division with division leading to two parabasal, or two basal cells or one parabasal and one basal cell (asymmetric replacement) 12. This results in two distinct HPV-induced pathways, as only parabasal cells can undergo terminal differentiation necessary to perform virion production (virion producing pathway) 15, whereas remaining basal cells retain the ability of division needed to establish an HPV induced transformation 16. When an infected basal cell starts dividing, a clonal population could arise, and each of these cells would contain a same number of HPV DNA copies. This clonal population can in time be transformed by the presence of viral oncoproteins (transforming pathway) 17. In our model asymmetric replacement is possible, implying that both pathways can occur simultaneously, in which one of the daughter cells remains at the basal cell layer retaining the ability to divide, and the other daughter cell becomes parabasal and acquires the possibility to differentiate and commence viral replication. Because in this scenario one basal daughter cell prevails, the next cell division can again result in an asymmetric epithelial cell replacement, making serial virion producing episodes (VPEs) possible. Each asymmetric replacement should give rise to a brand new batch of virions, measurable as a surge in serial type-specific viral load measurements, as long as infected basal cells remain. In the transforming pathway, an initial clonal population build up occurs because the infected basal cells retain the possibility of division, and if unchecked or unchallenged by immunity leads to CIN3+. Because of the clonal nature and the constant amount of HPV DNA per clone a linear increase of type-specific viral load is observed (viral loads in logarithmic scale on *y*-axis against time on a linear scale expressed in days on *x*-axis) in serial measurements (*R*² > 0.9 and slope = 0.003 HPV copies/cell per day) 7. Regression of a clonal population would then result in a linear decrease in type-specific viral load in time.

In this study, we wanted to investigate if serial viral load measurements can differentiate between regressing lesions and serial virion producing transient infections in women with persistent type-specific HPV infections.

We retrospectively calculated slopes between viral load measurements in untreated women with long-term type-specific HPV DNA positivity to try to define regressing lesions and serial transient infections with latency based on linearity of measurements and differences in slopes, and to see if these findings are in agreement with the proposed asymmetric epithelial cell replacement model.

## Materials and methods

### Samples for HPV analyses

This retrospective natural history study was set up using the RIATOL cervical cancer screening and diagnostic follow-up database. The laboratory for clinical and molecular pathology (RIATOL), Sonic Healthcare, Antwerp, Belgium is processing more than 100,000 liquid-based cytology (LBC) samples annually, representing approximately one quarter of women participating in cervical cancer screening in Belgium. Since June 2006, all LBC samples were subjected to a density gradient to remove free virions (BD Diagnostics, Burlington, SC) and the enriched cell pellet (*n* = 781,737 samples from 359,410 women) was subjected to HPV measurements to enable HPV-guided cytology 18. Therefore, cytology is not considered an independent parameter and was not used in this study. Cervical samples were collected in the framework of cervical cancer screening. In Belgium, costs for collection and interpretation of Pap smears are partially reimbursed. This was carried out without interval constraint until mid-2009 and, since then, on biennial basis till end 2012, and since 2013 on triennial basis for screening unless previous samples were abnormal. Local follow-up guidelines for management of cervical abnormalities are in agreement with EU guidelines 19,20. The RIATOL cervical cancer screening and diagnostic follow-up database contains all the cytologic, histologic, treatment, and virologic data, which are linked using a unique subject identification number. We selected untreated women in which persistent type-specific HPV DNA was detected in at least three consecutive viral load measurements, with the last smear taken between June and December 2013. Women with transient infections (±0.3 HPV copies/cell per day) or progressing lesions (*R*² > 0.9; +0.003 HPV copies/cell per day) were excluded 7. All data for this retrospective study were anonymized, no additional tests were performed outside routine practice, and there was no cost or additional risk for patients.

### Type-specific quantitative HPV measurement (qPCR assay)

A clinically validated real-time quantitative PCR 10 was used to amplify 18 HPV types: HPV6E6, 11E6, 16E7, 18E7, 31E6, 33E6, 35E6, 39E7, 45E7, 51E7, 52E7, 53E6, 56E7, 58E7, 59E7, 66E6, 67L1, and 68E7 as previously described by Micalessi et al. 21. A *β*-globin real-time quantitative PCR was used to assess the DNA quality and to estimate the number of cells in the test sample 21. This *β*-globin control PCR was considered positive when at least 1000 cells could be measured 10. The analytical sensitivity of the different type-specific HPV qPCR’s varies between 1 and 100 HPV copies/reaction 22. The number of HPV copies was divided by the number of cells to calculate the viral load (HPV copies/cell), and the threshold of positivity was 0.0001 HPV copies/cell.

### Statistical analysis

#### Calculation of coefficient of determination (*R*²) and slope

For each subject, the successive viral loads (HPV copies/cell) of each individual HPV type were plotted on a logarithmic scale (*y*-axis) against time on a linear scale expressed in days (*x*-axis). In six women were multiple HPV types were detected simultaneously each HPV infection (*n* = 15) was analyzed separately.

The slopes between two successive measurements were defined as log10(type-specific HPV load on date 1) −log10(type-specific HPV load on date 2) divided by the number of days occurring between date 1 and date 2 7.

For three or more consecutive measurements, the slope was calculated between the consecutive viral load measurements using a simple linear regression model [*y* = *a* + *bx*, where *y* is the predicted log10 (viral load), *a* is the intercept, *x* is the time interval, *b* is the slope (change in log10 viral load per unit of time)]. For each regression, the coefficient of determination *R*² was calculated, which is a measure of deviation between the regression line and the observed points. We considered a *R*² of ≥0.85 as a linear increase or decrease, representing the doubling or halving of a clonal population with a constant amount of HPV DNA per clonal cell. For regressing infections the viral halving time (*V*_HT_) in days was calculated for each HPV type by (ln 2)/slope. The latency boundary was only calculated when serial measurements were nonlinear (*R*² < 0.85) by selecting the highest and lowest measured viral load and adding or subtracting 0.5 log HPV copies/cell.

#### Calculation of the number of VPEs in serial transient infections with latency

The type-specific viral doubling time (*V*_DT_) calculated in clonal populations that evolved to CIN3+ 7 is representative for how much time (days) it takes for this clonal population (with a constant amount of HPV DNA copies/clonal cell) to double. We used this type-specific *V*_DT_ period as a proxy for the time needed for asymmetric basal cell division resulting in latency and serial virion productions. To calculate the number of VPEs in the latency group (*R*² < 0.85), the number of days a specific HPV type is detected was divided by the number of days of the corresponding type-specific *V*_DT_. For HPV 11, 53, and 68 an average *V*_DT_ of 286.3 days was used to calculate the number of VPE. For all other HPV types, the type-specific *V*_DT_ was used 7.

The calculated *R*² and slopes were compared between cases with serial transient infections and regression cases using the MedCalc® program (MedCalc Software, Oostende, Belgium) 23. For abnormally distributed variables, median values are given. To compare differences in slopes between serial transient infections with latency and regression a two-sided Mann–Whitney *U* test was used.

## Results

### Long-term detection of type-specific HPV DNA

A database search identified 261 untreated women with a smear between June and December 2013, which had a persistent type-specific HPV DNA detected (270 infections) in at least three of their last smears for a mean period of 1156 days (95% CI 1074–1238 days). The median age of women in the whole group was 39.0 years (95% CI 37.0–41.0 years). There was no statistical difference in duration in which the different HPV types were detected. On average the untreated women had five smears within this period (Table1).

**Table 1:** Persistent HPV-type-specific detection in three or more consecutive measurements in untreated women, subdivision in regression, and serial transient infections with latency based on *R*²

All					Latency group (*R*² < 0.85)						Regression (*R*² ≥ 0.85)					
HPV type	*n*	Mean number of days detected	Range (days)	Average number of smears	*n*	%	Days	*R*²	Slope (absolute)	VPE (*n*)	*n*	%	Days	*R*²	Slope	V_HT_ (days)
6	6	1313	550–2077	5	6	100	1314	0.3166	0.00051	5	0	0				
11	3	1116	714–1800	6	3	100	1116	0.1689	0.00024	4	0	0				
16	47	1042	865–1218	4	25	53.2	1393	0.1910	0.00034	5	22	46.8	642	0.9669	−0.00425	163
18	8	944	395–1492	4	5	62.5	1161	0.2402	0.00101	3	3	37.5	583	0.9652	–0.00577	120
31	48	1382	1162–1602	5	24	50.0	1887	0.1234	0.00052	6	24	50.0	876	0.9595	–0.00408	170
33	9	975	787–1164	4	1	11.1	1329	0.0072	0.00025	5	8	88.9	931	0.9592	–0.00296	234
35	9	1039	708–1370	4	3	33.3	1054	0.3109	0.00036	4	6	66.7	997	0.9701	–0.00303	229
39	15	998	600–1395	5	7	46.7	1485	0.1490	0.00028	5	8	53.3	571	0.9765	–0.00458	151
45	6	1117	634–1601	4	4	66.7	1395	0.3450	0.00009	4	2	33.3	562	0.9551	–0.00265	262
51	12	1158	756–1559	5	6	50.0	1566	0.1503	0.00088	6	6	50.0	750	0.9733	–0.00245	283
52	19	1054	757– 1351	5	10	52.6	1475	0.1493	0.00055	6	9	47.4	587	0.9802	–0.00202	343
53	21	1368	1025–1711	6	15	71.4	1632	0.2482	0.00061	6	6	18.6	705	0.9708	–0.00488	142
56	13	1533	1052–2013	6	10	76.9	1832	0.1444	0.00039	10	3	23.1	535	0.9410	–0.00483	144
58	15	1213	783–1643	5	8	53.3	1716	0.1009	0.00040	7	7	46.7	638	0.9675	–0.00409	170
59	12	1161	552–1770	5	5	41.7	2170	0.2408	0.00069	9	7	58.3	440	0.9724	–0.00396	175
66	9	732	467–998	4	2	22.2	1032	0.4249	0.00102	5	7	77.8	646	0.9696	–0.00564	123
67	10	1035	528–1543	5	2	20.0	2187	0.1018	0.00017	8	8	80.0	748	0.9468	–0.00390	178
68	8	813	445–1182	4	7	87.5	901	0.3958	0.00096	3	1	12.5	201	0.9935	–0.00480	144
All	270	1156	1074–1238	5	143	52.6	1553	0.1977	0.00012	5	127	47.4	708	0.9658	–0.00330	210

*R*², coefficient of determination; *n*, number of infections; slope (absolute), average absolute slope calculated between two consecutive measurements; VPE, virion producing episode (*n*); slope, calculated between three or more measurements by linear regression (*R*² ≥ 0.85); V_HT,_ viral halving time (ln2/slope).

**Figure 2:**
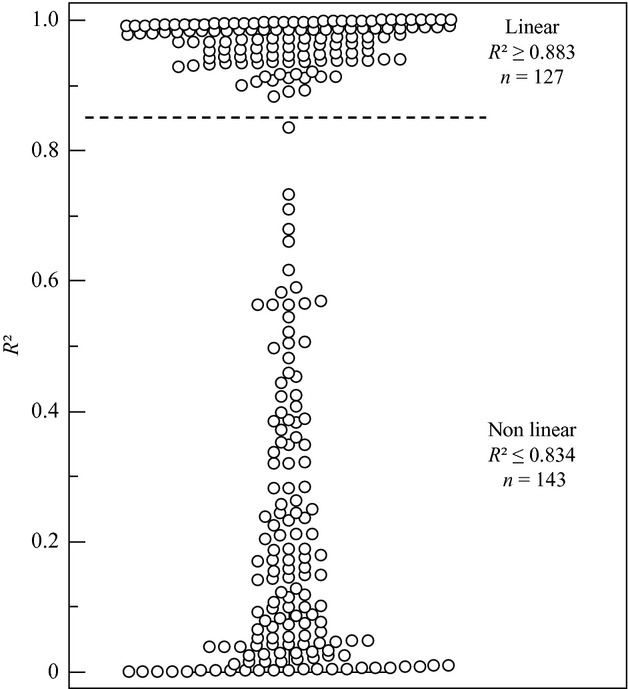
Subdivision in Linear (*R*² > 0.85) and non-linear (*R*² < 0.85) processes based on calculation of *R*² using 3 consecutive type specific viral load measurements. Dashed line *R*² = 0.85.

Using *R*² calculated with the viral load of the three last smears, the whole group can be subdivided in a group with a linear viral load course (*R*² ≥ 0.8827) (*n* = 127) and a group with a nonlinear viral load course (*R*² ≤ 0.8340) (*n* = 143) (Fig.2). The median *R*² in the group with linear type-specific viral load course was 0.9733 and 0.1270 in the nonlinear group.

In the group with a linear viral load course (*R*² ≥ 0.85), the median slope over three or more measurements was always decreasing (regressing group; −0.0033 HPV copies/cell per day; 95% CI −0.0041 to −0.0027). For the group with a nonlinear course, only slopes between two consecutive measurements were calculated. In this group, only a limited increase or decrease was measured with slopes between 0.0010 and −0.0010 HPV copies/cell per day (latency group). The HPV type-specific characteristics in the latency and regression groups are shown in Table1. Difference in slopes between the two groups was highly significant (*P* < 0.0001).

Comparing the HPV types detected in regression and in latency groups showed HPV type-specific preferences. In untreated women no regressing patterns could be detected for HPV types 6 and 11. Whereas serial transient infection patterns were detected for all HPV types tested. HPV types 67, 33, 66, 35, and 59 preferentially produce lesions that regress, whereas HPV types 6, 11, 68, 56, 53, and 18 rather produce infections with serial VPEs. For HPV types 16, 31, 39, 51, 52, and 58 the number of regressing and serial VPEs was approximately the same.

Follow-up data (from January 2014 until October 2014) were available for 199 cases (73.7%), of which 110 from the latency group and 89 from the regression group. An overview of the follow-up outcomes is given in Table2. There were significantly more women that cleared their HPV in the regression group 65.7% (44/67) than in the latency group 25.7% (19/74) (*P* < 0.0001). There were significantly more CIN 2 cases detected in follow-up in the latency group 18.9% (14/74) than in the regression group 3.0% (2/67) (*P* = 0.0068). In the latency group, one case of invasive cancer was detected, and there were significantly more CIN2+ cases 23% (17/74) compared to the regression group 6.0% (4/67; *P* = 0.0095).

**Table 2:** Follow-up outcomes in serial transient infections with latency and regression

	Latency group		Regression group		*P*-value
	*n*	%	*n*	%	
Cytology and HPV follow-up negative	19	25.7	44	65.7	<0.0001
CIN 0	23	31.1	11	16.4	=0.0657
CIN 1	15	20.3	8	11.9	NS
CIN 2	14	18.9	2	3.0	=0.0068
CIN 3	2	2.7	2	3.0	NS
Invasive cancer	1	1.4	0	0	
Total follow-up	74	100.0	67	100.0	NS
HPV not cleared	36	25.2	22	17.3	NS
No histology/follow-up	33	23.1	38	29.9	NS
Total	143		127		

Last of (≥3) measurements between June and December 2013, follow-up until October 2014. CIN, cervical intraepithelial neoplasia; NS, not significant; *n,* number of infections.

**Figure 3:**
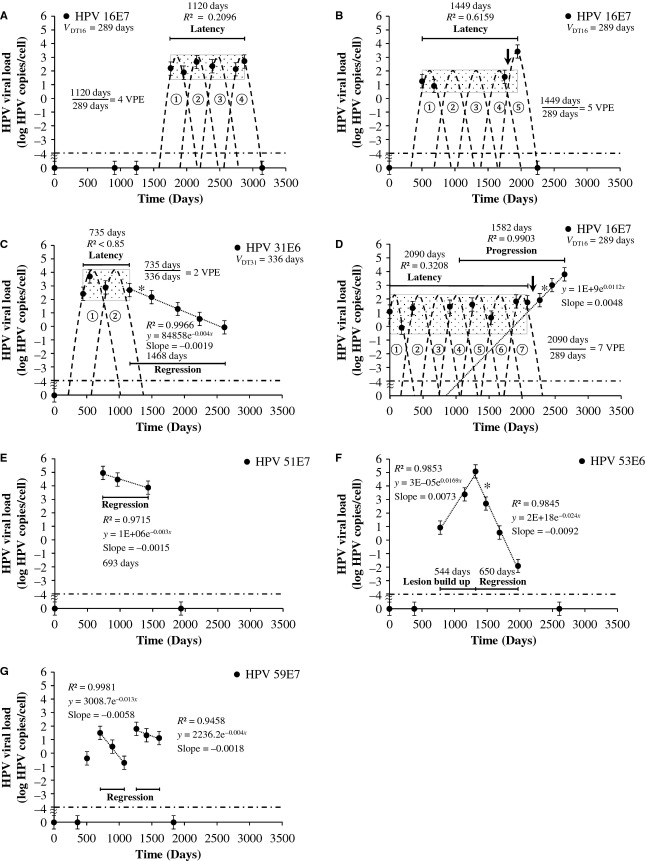
Representative cases showing the different viral load patterns in latency and regression. (A) Serial virion producing episodes with latency (B) Serial virion producing episodes with latency (exit scenario 1) (C) latency followed by regression (exit scenario 2) (D) Latency followed by progression (E) regression (F) progression or lesion build up followed by regression (G) Serial regression of clonal populations. Dash-dotted line, threshold of positivity (0.0001 HPV copies/cell); *V*_DT_, median viral doubling time in single type infections leading to CIN3+ 7; Number of virion producing episodes (VPE) in latency (*R*² < 0.85) = period of type specific HPV DNA detection (days)/HPV type specific VDT (days); dashed line, virion producing episode; arrow: biopsy CIN 0; dotted rectangle, latency threshold; dotted line, progression or regression (*R*² > 0.85); *hinge 7. Slope in HPV copies/cell per day.

The representative cases of the different serial viral load patterns observed in serial transient infections with latency and regression are shown in Figure3.

## Discussion

The aim of this study was to define regressing lesions and serial transient infections with latency based on serial type-specific viral load measurements.

The extensive data presented in this large retrospective study are in agreement with the proposed asymmetric epithelial replacement model. This model also allows simultaneous occurrence of regression of lesions as well as serial VPEs. We could clearly subdivide the group of untreated women with persistent type-specific HPV DNA detected over a longer period of time in two subgroups based on *R*², using the type-specific viral load in the last three consecutive measurements. In the group with *R*² ≥ 0.85 the viral load decreased with 0.0033 HPV copies/cell per day (slope = −0.0033). This negative slope clearly represents a halving of a clonal population of basal cells with a constant number of HPV DNA copies per clonal cell. So far, in the available follow-up data (10 months), the majority (65.7%; 44/67) of these women had a normal cytology with negative HPV test after a regressing viral load period. This might suggest that the treating physicians decided to wait and see as the type-specific viral load was dropping in three consecutive smears, independently of the cytology results. In the group with *R*² < 0.85, however, the differences in slopes was only minimal and probably reflects a serial production of a constant virion amount. A serial virion production would imply that these women are probably cyclically infectious over a longer period of time. This is in contrast with women with regressing lesions who are not infectious as infected basal cells do not allow production of new virions. Furthermore, during serial transient infections with latency, the remaining latent basal cell can still divide, and each new generation of basal cells have more risk to be transformed under the influence of the sustained oncogenic E6/E7 protein production. This increased risk for development of CIN in serial transient infections with latency is illustrated in the more severe histology outcome of 23.0% CIN2+ in this group (*P* = 0.0095). Delayed action by clinicians was probably induced by a combined lack of persistent abnormal cytology and absence of elevated or increasing type-specific viral loads. During serial virion production effects on cytology would only be minimal as desquamating cells have a limited time span, and the accumulation of newly formed virions within the cell cytoplasm is cytologically well recognizable as low-grade squamous intraepithelial lesions (L-SIL).

Clearing of the HPV infection then becomes possible in two different scenarios.

In exit scenario 1, depletion of parabasal cells occurs. When the last HPV-infected basal cell division occurs double the amount of parabasal cells (two) begin differentiation and start an ultimate virion production. This results in double the number of virion producing cells compared to the latency period in which asymmetric division only produces one parabasal cell, and results in a higher measured load compared to the latency period. In some of the cases with serial transient infections with latency a higher load is seen before clearing HPV (Fig.3B). However, detection of a double amount of HPV load is problematic, as with our current method only a 1 log difference in viral load is statistically significant 21. When, because of the asymmetric HPV-infected basal cell division, the virion producing and HPV onco-protein transforming pathways occur simultaneously (same HPV type), this has an impact on detection of underlying progressing lesions in serial measurements. Parameters influencing detection of an underlying transforming process are the latency boundary due to virion production, which has a variation even for a same HPV type in the upper limit thresholds (Fig.3A, B, and D), and the viral doubling time (*V*_DT_) of this underlying clonal population 7.

In the second exit scenario 2, a depletion of basal stem cells occurs (Fig.3 C, E, F, and G). In this study, we also encountered cases where a serial transient infections with latency period was followed by a linear regressing period or progressing period (Fig.3 C and D) illustrating the dual pathway after initial HPV infection of a single basal cell. The differences between basal and parabasal cells probably lies in the limited amount of times parabasal cells can divide before starting differentiation and desquamation, whereas basal cells can keep on dividing and do not desquamate. Therefore, basal cell division or more correctly the clonal expansion defines size and growth of nonvirion producing dysplastic lesions. These basal cells also represent a reservoir of potential virion producing cells, when after cell division these daughter cells differentiate and allow new virus production 15. Parabasal cell division on the other hand defines the size and duration of virion productive infections/lesion that are limited in time.

Using serial HPV type-specific viral load measurements, we could for the first time identify regressing CIN2 and CIN3 lesions. Regression of the clonal population of HPV-infected basal cells could be due to cytotoxic T-cell response. Not surprisingly, regression is a slow process because HPV infection causes no viremia, evades innate immune response and delays activation of adaptive immunity 24. This is in agreement with our data, with an average calculated viral halving time of 210 days, and a prolonged period of HPV type-specific persistence of almost 2 years (708 days), before the majority of women clear their HPV infection without treatment.

Serial measurements may have the disadvantage that at least three sampling points are needed to be able to calculate linearity of viral load measurements, but allows measurement of regressing lesions and calculation which of the two HPV induced pathways is occurring. It is thus not a risk assessment but rather a mathematical representation of the virion producing or basal cell transforming pathway. The advantage of using HPV type-specific serial viral load measurements is that it can be used to triage each separate HPV infection into a clonal or nonclonal process, irrespective of the age of the woman, cytology result and number of simultaneously occurring HPV infections. Furthermore, by calculating *R*² and slope each type-specific HPV infection in HPV positive women can be categorized in one of four HPV courses (clonal progressing 7, clonal regressing, transient 7, and serial transient infections). Because only progressing clonal processes lead to cancer, all other processes are or transient or lead to regression. Even in women with multiple HPV infections the HPV infection that has a clonal course can be identified. Beside identifying the clonal process (*R*² ≥ 0.85), the slope indicates if the measured process is progressing (+0.003 HPV copies/cell per day) or regressing (−0.003 HPV copies/cell per day). Because the majority of clonal processes regress, triaging HPV infections by *R*² and slope could substantially reduce biopsy taking and overtreatment of regressing lesions. Women with single or with multiple HPV infections in which no clonal progressing process was detected would then need a less stringent follow-up. Noninfectiousness of regressing lesions would be another argument to the wait and see option. Prospective studies are needed to confirm the efficacy of triaging HPV infections by *R*² and slope in cervical cancer screening.

We conclude that serial type-specific viral load measurements allows discrimination between serial transient infections with latency or regressing lesions in women who have prolonged type-specific HPV persistence, and that the evolution of the viral load is an objective measurable indicator of the natural history of HPV infections and could be used for future triage in HPV-based cervical screening programs.
